# New York Letter

**Published:** 1895-08

**Authors:** 


### NEW YORK LETTER.

NEW YORK, July 31, 1895.

Dr. George B. Fowler, a well-known physician, has been appointed Commissioner to the Board of Health in the place of Dr. Cyrus Edson, who resigned the position last month.

Other changes have occurred this month in the Board of Health. Dr. John T. Nagle, the Register of Vital Statistics, was retired, at his own request, on a pension of $600 a year. Dr. Nagle has been connected with the Board of Health for twenty-six years, and has held the position of Register since '87. Dr. Roger S. Tracy, the Deputy-Register, has been appointed Register in Dr. Nagle's place, a position which he had held previous to '87, but the office being a political one, he was deposed at that time and made Deputy-Register, the fact that he had been very efficient not weighing in his favor. Now that he has been reappointed to his former position, it is to be hoped that he will be allowed to keep it.

On July 1st, Dr. Buchanan was executed for the murder of his wife, Annie Sutherland. It is rare for a medical man to figure as a criminal, and the detection of Dr. Buchanan's crime, his subsequent trial and the able defense by his lawyers, Mr. Jerome and Dr. O'Sullivan, form an unusual and interesting chapter in the annals of criminal trials. The trial is said to have cost the State $30,000. After his conviction and after sentence of death had been pronounced by Recorder Smyth, in April, '93, unusual efforts were made by his lawyers to save him, and every technicality was called into play. A new trial was asked on the ground of incapacity of one of the jurors, and Recorder Smyth delayed his decision until August, then denied the application and fixed October 2d, '93, as the date of execution. The case was appealed, but the Court of Appeals sustained the verdict in February of this year, and he was resentenced twice after that date; finally the Court of Appeals resentenced him in May to be executed during the week beginning July 1st.

Dr. Buchanan was a Scotchman and began life as a drug clerk in Nova Scotia. He afterwards studied medicine in Chicago, obtaining a degree in '83. He was married and went to

Edinburgh to study, and in '87 settled in New York. He was not very successful, became discouraged, ran into debt and drank. His wife left him and he obtained a divorce from her. His second wife, who was ten years his senior, a woman of bad reputation, but possessed of some means, he met later and married in 1890. She had made a will, leaving her money to him, and died in April, '92, after a short illness. The physician who was called in gave a certificate of death from apoplexy. Buchanan left New York and within a month was remarried to his first wife and returned with her to New York, where they were living under an assumed name. Suspicion was directed towards him from various remarks made to associates, and June 5th of that year, the body of his second wife was exhumed, examined, and Buchanan was arrested on a charge of murder the following day, and his trial began in March, '93. It was through the efforts of a newspaper that this was brought about.

He was executed by electricity, which was rapidly and painlessly performed. A voltage of 1,740 was turned on for four seconds and then the current was reduced to four hundred volts for thirty seconds.

The autopsy showed extravasation of blood into the brain and internal organs, especially the liver, showing conclusively that death occurred in the electric chair.

The newspapers have for some time been trying to stir up controversy and make capital out of unwarranted assertions that those executed do not die in the chair but are killed by the autopsy knife. Dr. A. H. Goelet, of this city, has contributed two able articles on "Execution by Electricity," one published in the *Electrical World*, February 16th, '95, and the other in the *American Medico-Surgical Bulletin*, April 1st, '95, completely confuting these assertions, and yet the *New York Sun*, of July 22d, contains a very sensational article headed: "Electricity Didn't Kill—Secrets of the Death Chamber at Auburn Prison," purporting to be an interview with a Syracuse physician, who has desired to try his method of resuscitation of those overcome by electricity upon those executed in this manner. He has at various times applied to the Governor for permission to try it, which has rightly been refused, and although not present at the Buchanan execution, he makes the absurd claim that

Buchanan would have recovered consciousness after the first application of the current had he been let alone and no remedies need have been applied to resuscitate him, and that after the second application, simple means would have served to restore consciousness without even using the bellows which this doctor has invented. And yet, a skilled electrician, only the other day, was killed while examining some lights which were out of order, coming in contact with an electric light current, and none of the methods applied to save him were successful.

As a matter of fact, electrocution has come to stay. It is absolutely painless for the reason that the current travels faster than the nerves can convey the painful impression, and this mode of execution is far more humane and scientific than hanging.

The other day the New York medical world was startled by the bold robbery in broad daylight of a Brooklyn physician, who was summoned by telephone and directed to an unoccupied house, and who, on answering the summons and entering the house, was set upon by two powerful men, gagged, bound and robbed of his watch, jewels and money. The tale was so startling and almost so improbable that the authorities were inclined to disbelieve it, until a couple of days later his watch and jewels were found in a well-known pawn-shop in New York City.

Another Brooklyn physician comes forward and tells how he was fortunate enough to escape a similar fate, being called to a tenement house, and fortunately met at the door by a woman who told him that the tenement he was looking for was vacant, and as he was leaving, a man stopped him and desired to conduct him to the supposed patient, but the doctor, becoming suspicious, refused to go, and returned home.

This is a danger to which doctors are exposed, and yet they may and do go about in the worst portions of the city at all hours, and are never molested. It would seem that the criminals are hard pushed when they decoy a physician by bringing him on an errand of mercy.

